# Supplementary material for: Secondary lymphoid organ fibroblastic reticular cells mediate trans-infection of HIV-1 via CD44-hyaluronan interactions
Source: Nat Commun. 2018 Jun 22;9:2436. doi: 10.1038/s41467-018-04846-w (PMC6015004; doi:10.1038/s41467-018-04846-w)
Supplement: Supplementary file 1 — Supplementary Information [file 41467_2018_4846_MOESM1_ESM.pdf]

Supplementary information

Secondary lymphoid organ fibroblastic reticular cells mediate trans-infection of HIV-1 via CD44-hyaluronan interactions

Murakami et al.

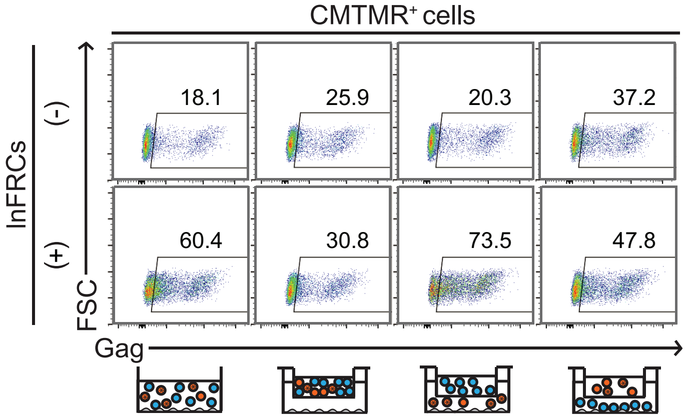

Supplementary Figure 1. FRCs isolated from lymph nodes enhance HIV-1 spread among donor T cells in a manner dependent on T cell-FRC contacts. Flow cytometry analysis of HIV-1 infection among $CMTMR^+$ cells in the presence or absence of lnFRCs and transwell inserts. The analysis of HIV-1 infection among $CMTMR^-$ cells in the same cocultures is shown in Fig. 1C.

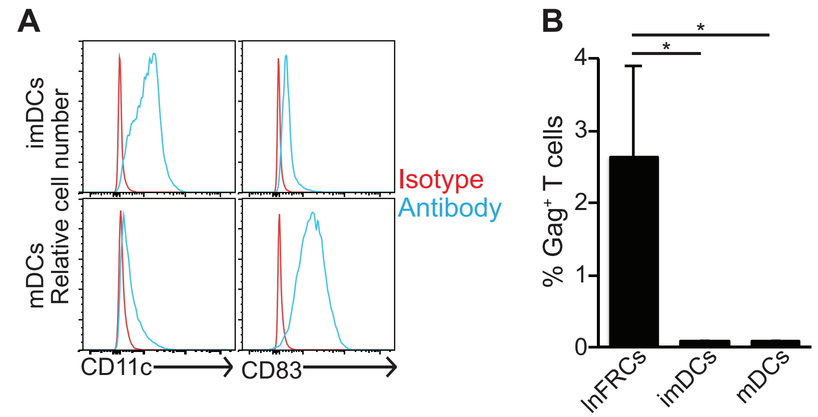

Supplementary Figure 2. Comparison of trans-infection mediated by FRCs with immature and mature DCs. (A) Flow cytometry analysis of cell surface marker expression on immature DCs (imDCs) and mature DCs (mDCs). imDCs were CD11c^{high} and DC-SIGN^{+} (see Fig. 4A), whereas mDCs were CD11c^{low}, CD83^{high} and CD169^{+} (see Fig. 4B). (B) The comparison of trans-infection mediated by lnFRCs with that by imDCs and mDCs. lnFRCs, imDCs and mDCs generated from monocytes isolated from peripheral blood of three different donors were cultured in the presence of 7.6 ng p24 of HeLa-derived virus for 2 h, washed extensively, and cocultured with A3.01 T cells for 6 days. A3.01 T cells were examined for expression of Gag by flow cytometry. Data represent the mean ±SD of two independent experiments performed with lnFRCs isolated from three different donors and imDCs and mDCs generated from monocytes isolated from peripheral blood of three different donors. The *p* values were determined using Tukey's test following one-way ANOVA. *, $p<0.05$.

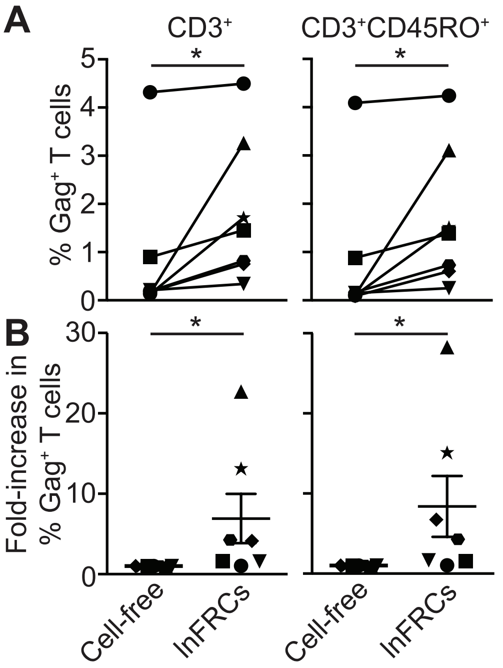

Supplementary Figure 3. FRCs isolated from lymph nodes promote HIV-1 infection of PBLs via trans-infection. lnFRCs were cultured in the presence of HIV-1_{NL4-3} (100 ng p24) for 2 h, washed twice, and cocultured with PHA-stimulated peripheral blood lymphocytes (PBLs) for 4 days. For the cell-free condition, PHA-stimulated PBLs were inoculated with the same amount of input virus for 2 hr, washed with RPMI-10 twice and cultured for 4 days. (A) Gag expression in $CD3^{+}CD45RO^{+}$ and $CD3^{+}CD45RO^{-}$ cells were analyzed by flow cytometry. Data represent the results of experiments performed using seven different pairs of lnFRCs and PBLs. lnFRCs derived from three different donors were paired with PBLs isolated from one of seven different donors. (B) Fold-increases in % Gag^{+} T cells. The mean ±SEM is shown. The p values were determined using Wilcoxon matched-pairs signed rank test. *, $p<0.05$.

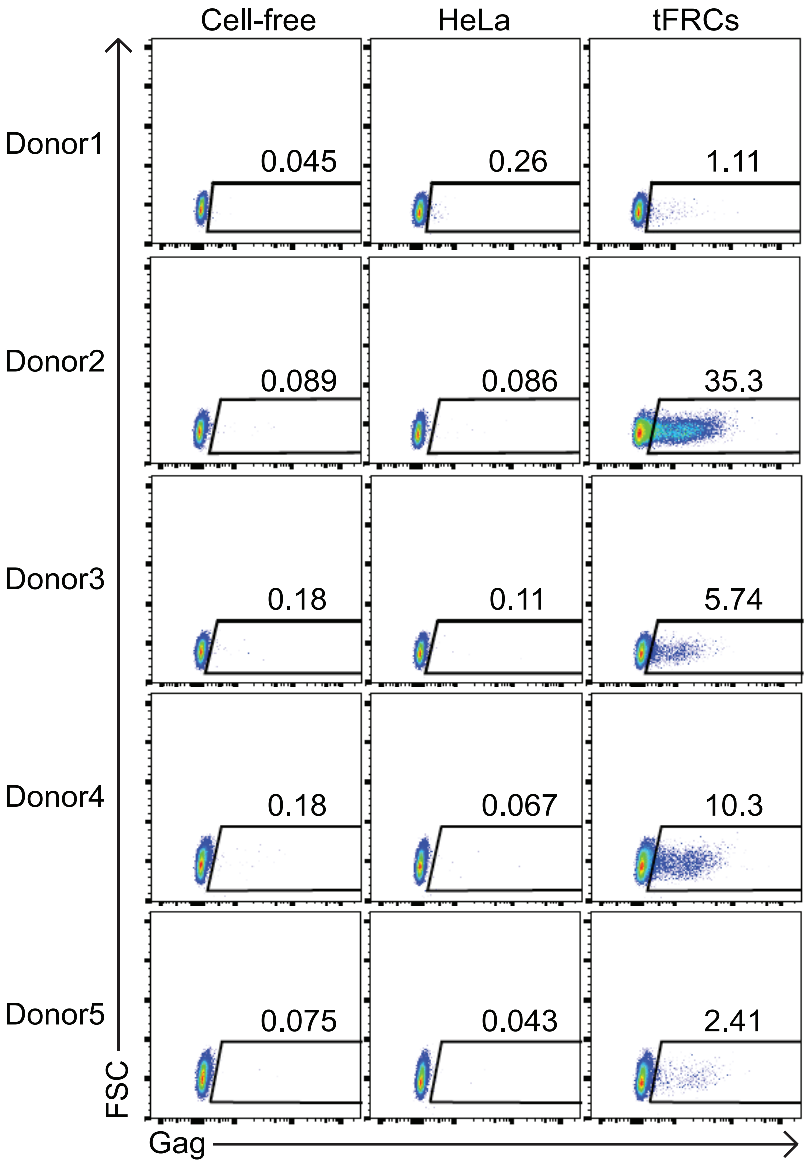

Supplementary Figure 4. FRCs isolated from tonsils mediate trans-infection of virus produced in donor-matched HLACs. Flow cytometry analysis of trans-infection of HLAC-derived virus to A3.01 T cells mediated by HeLa cells and tFRCs is shown. tFRCs and HLACs were prepared from the tonsils of the same donor in each experiment. Data represent the results of independent experiments performed using tFRCs and HLACs from five different donors. The set of results obtained by using Donor4 tonsillar cells is shown in Fig. 3B.

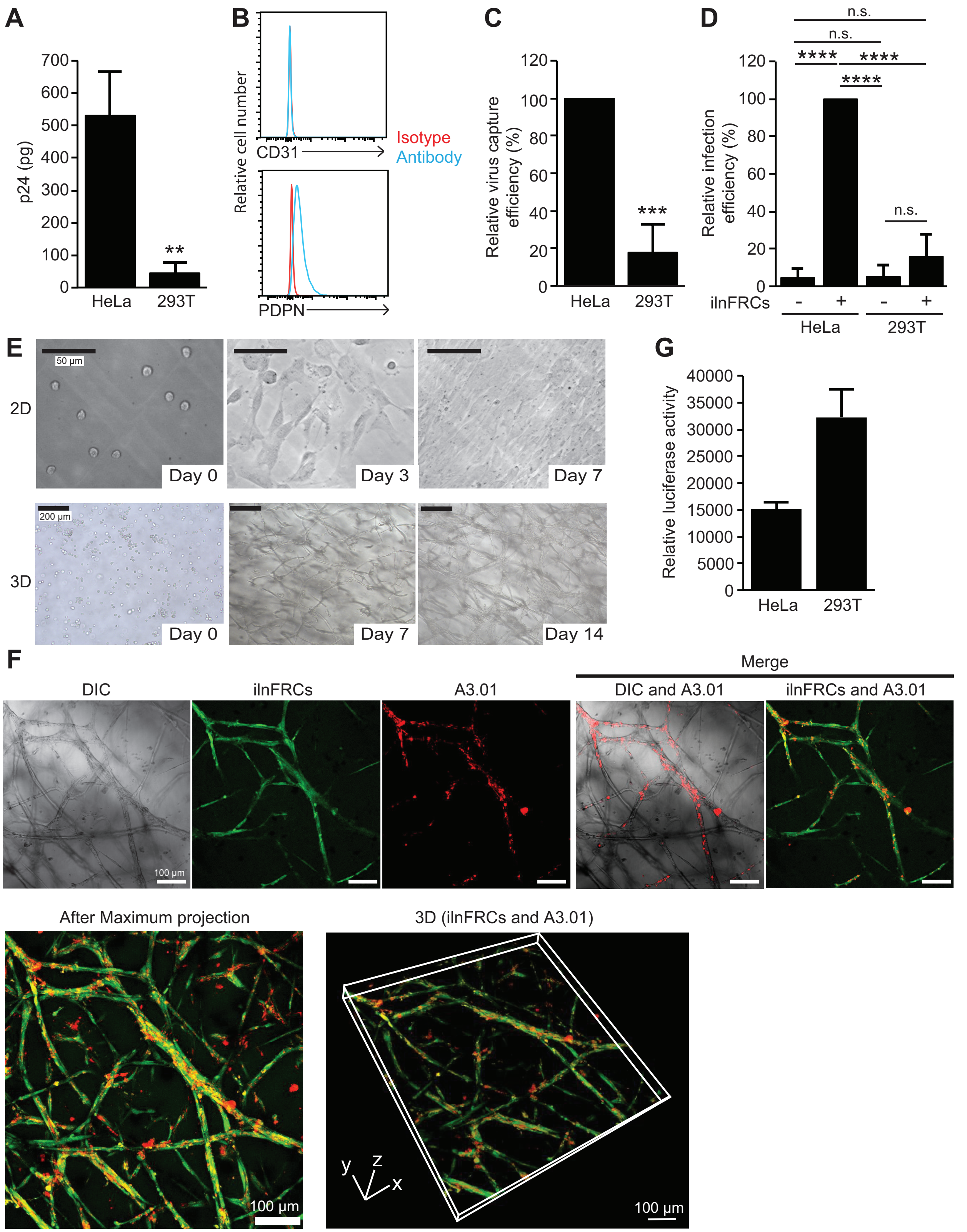

Supplementary Figure 5. FRCs mediate trans-infection of HIV-1 produced by HeLa cells

more efficiently than HIV-1 produced by 293T cells in 2D and 3D culture systems. (A) Quantification of the amount of HeLa- and 293T-derived HIV-1_{MJ4} captured by lnFRCs. lnFRCs isolated from three different donors were used. (B) Flow cytometry analysis of CD31 and PDPN expression on the surface of lnFRCs immortalized by transfection of HPV E6 and E7 (ilnFRCs). (C) Quantification of the amount of HeLa- and 293T-derived HIV-1_{NL4-3} captured by ilnFRCs in 2D cultures. For inoculation, 7.6 ng p24 was used. (D) Comparison of ilnFRC-mediated trans-infection of A3.01 T cells between HeLa- and 293T-derived viruses in 2D cultures. (E) Micrographs of ilnFRCs cultured in a conventional tissue culture plate (2D; scale bar, 50 μm) or in PEG gels (3D; scale bar, 200 μm) over the course of up to 7 days (2D) or 14 days (3D) are shown. (F) T cells and ilnFRCs cocultured in the 3D system. ilnFRCs encapsulated in a PEG gel and A3.01 T cells were stained with fluorescent dyes, CMFDA (green) and CMTPX (red), respectively. The stained A3.01 T cells were added to the gel and cultured for 3 days. The 3D coculture was examined using a confocal microscope. Upper panels show images obtained from a single focal plane. The lower left panel shows a maximum projection image, and the right panel shows a 3D reconstructed image. Note that T cells penetrate into the PEG gel-based 3D cultures and form contacts with ilnFRC networks therein. Scale bar, 100 μm. (G) Relative infectivity of HeLa- and 293T-derived virus stocks used for experiments shown in Figure 5F was determined by TZM-bl assay using the same amount (based on the p24 amount) of viruses. The Y axis is in an arbitrary unit. Data represent the mean ±SD of triplicate wells of one pair of virus stocks. Another pair of stocks used for Fig. 5F showed a similar result. Data shown in panels A, C, and D represent the mean ±SD of three independent experiments. The *p* values were determined using two-tailed student's t-test for A and C and Tukey's test following one-way ANOVA for D. **, $p<0.01$; ***, $p<0.001$; ****, $p<0.0001$; n.s., not significant.

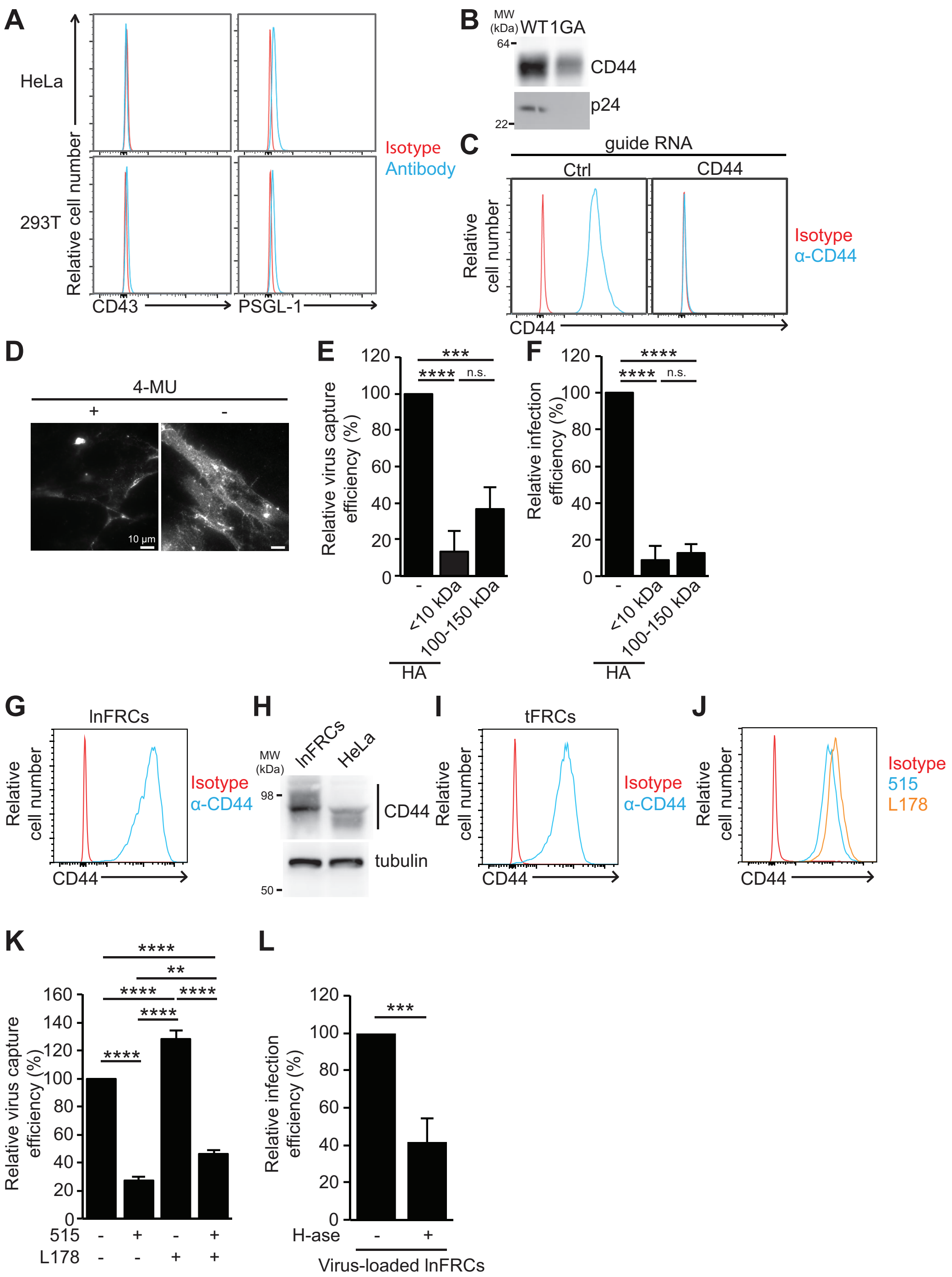

Supplementary Figure 6. lnFRCs mediate trans-infection of HIV-1 via the interactions between hyaluronan bound to CD44 on virus particles and CD44 expressed on the surface of lnFRCs. (A) Flow cytometry analysis of cell surface CD43 and PSGL-1 expression on indicated cell lines. (B) Pelletable materials in culture supernatants of 293T cells cotransfected with a plasmid expressing CD44 and a HIV-1 molecular clone encoding WT Gag or myristylation-deficient Gag (1GA) were examined by immunoblotting using HIV-Ig and anti-

CD44 antibody. Note that in the absence of HIV-1 particle release (1GA) as determined by the p24 release, extracellular CD44 release is limited unlike in the presence of particle release (WT). This observation indicates that the majority of extracellular pelletable CD44 is associated with released virus particles. (C) Flow cytometry analysis of cell surface CD44 expression on HeLa cells transfected with a plasmid expressing control or *cd44*-targeting guide RNA (gRNA) along with Cas9. (D) Detection of hyarulonan (HA) on the surface of lnFRCs using biotinylated HA binding protein. Note that the signal decreased markedly when cells were pretreated with 4-methylumbelliferone (4-MU) that inhibits hyaluronan synthesis. A representative result is shown for experiments performed using lnFRCs from three different donors. Scale bar, 10 μm (E) The effect of exogenous HAs on capture of HeLa-derived virus by lnFRCs. HA of two molecular weight ranges, <10 kDa and 100-150 kDa, were added at 2.5 mg/ml. lnFRCs were incubated with 7.6 ng p24 of HeLa-derived virus for 2 h in the presence of HAs and washed extensively. (F) The effect of exogenous HAs on lnFRC-mediated trans-infection of HeLa-derived virus in A3.01 T cells. Data represent the mean ±SD of three (E) and four (F) independent experiments in which lnFRCs from one donor were used. We obtained similar results by using lnFRCs isolated from three different donors. (G) Flow cytometry analysis of CD44 expression on lnFRC surface. A representative result is shown for analyses of lnFRCs from three different donors. (H) Western blot analysis of CD44 expression in lnFRCs and HeLa cells. A representative result is shown for analyses of lnFRCs from three donors. (I) Flow cytometry analysis of CD44 expression on the surface of tFRCs. A representative result is shown for analyses of tFRCs from five different donors. (J) Two anti-CD44 antibodies, clones 515 and L178, bind to lnFRC surface to a similar extent. (K) lnFRCs were pretreated with 10 μg/ml anti-CD44 antibodies (clone 515 or L178) or isotype control for 1 h. After removal of excess antibodies by washing, the cells were cultured in the presence of 7.6 ng p24 of HeLa-derived virus for 2 h. After extensive washing, the amount of virus captured by lnFRCs was quantified by p24 ELISA assays. (L) lnFRCs were cultured in the presence of 7.6 ng p24 of HeLa-derived virus for 2h. After extensive washing, the virus-loaded cells were treated with 10 units/ml hyaluronidase (H-ase) for 1h, washed extensively, and cocultured with A3.01 T cells for 6 days. A3.01 T cells were analyzed for expression of Gag. For panels K and L, data represent the mean ±SD of three independent experiments using lnFRCs isolated from three different donors. The p values were determined using Tukey's test following one-way ANOVA for G, H, K, and L. **, $p<0.01$; ****, $p<0.0001$; n.s., not significant.

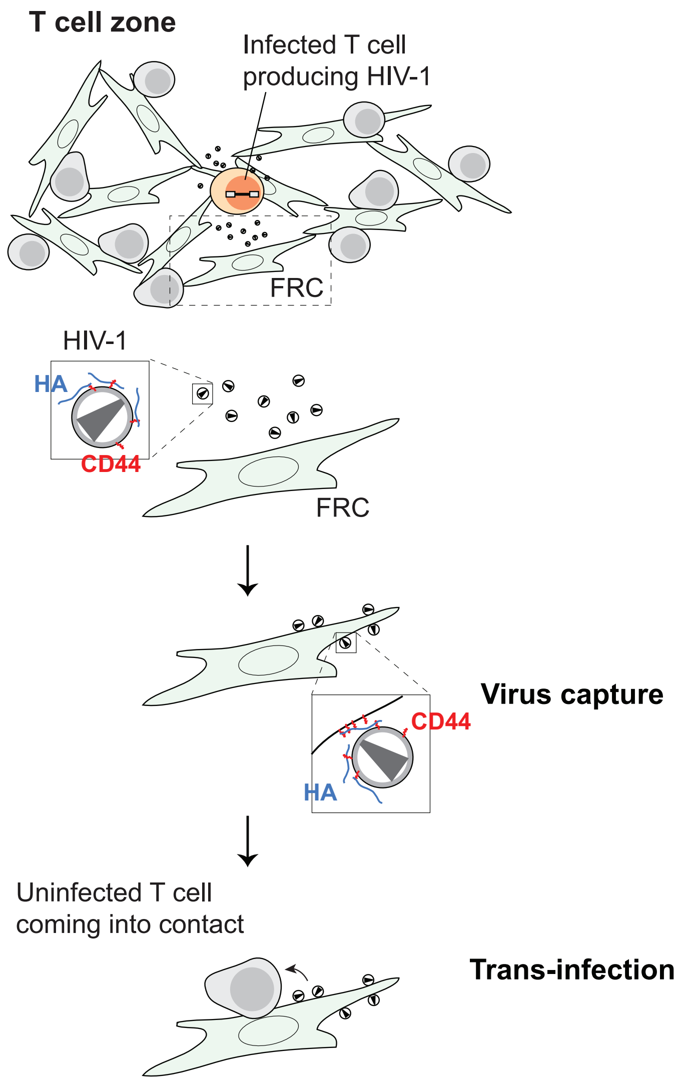

Supplementary Figure 7. A working model for FRC-mediated trans-infection of HIV-1. The 3D network of FRCs in T cell zones form frequent contacts with and serve as migratory tracks for T cells. Our findings support a model in which FRCs capture virus particles, either released by infected T cells in SLOs or delivered to SLOs from other infection sites, via the interactions between HA bound to CD44 on virus particles and CD44 on FRCs. These interactions can be prevented upon removal of HA from virus particles (by H-ase treatment of virus), dissociation of HA and virus-associated CD44 (by pretreatment of virus with anti-CD44 clone 515), or blocking of CD44 on FRC surface (by anti-CD44 clone 515 or excess HA) as shown in Figures 6 and 7. According to this model, FRCs transfer captured virus particles to target T cells when they contact, thereby contributing to HIV-1 spread in SLO T cell zones.
